# Ultrathin quasi-hexagonal gold nanostructures for sensing arsenic in tap water†

**DOI:** 10.1039/d0ra02750b

**Published:** 2020-05-27

**Authors:** Anu Prathap M. Udayan, Batul Kachwala, K. G. Karthikeyan, Sundaram Gunasekaran

**Affiliations:** Department of Biological Systems Engineering, University of Wisconsin Madison WI 53706 USA guna@wisc.edu

## Abstract

Monodispersed colloidal gold nanoparticles (AuNPs) were synthesized by an easy, cost-effective, and eco-friendly method. The AuNPs were mostly quasi-hexagonal in shape with sizes ranging from 15 to 18 nm. A screen-printed electrode modified with AuNPs (AuNPs/SPE) was used as an electrochemical sensor for the detection of As(iii) in water samples. The mechanistic details for the detection of As(iii) were investigated and an electrochemical reaction mechanism was proposed. Under the optimal experimental conditions, the sensor was highly sensitive to As(iii), with a limit of detection of 0.11 μg L^−1^ (1.51 nM), which is well below the regulatory limit of 10 μg L^−1^ established by the United States Environmental Protection Agency and the World Health Organization. The sensor responses were highly stable, reproducible, and linear over the As(iii) concentration range of 0.075 to 30 μg L^−1^. The presence of co-existing heavy metal cations such as lead, copper, and mercury did not interfere with the sensor response to As(iii). Furthermore, the voltammogram peaks for As(iii), lead, copper, and mercury were sufficiently separate for their potential simultaneous measurement, and at very harsh acidic pH it may be possible to detect As(v). The AuNPs/SPE could detect As(iii) in tap water samples at near-neutral pH, presenting potential possibilities for real-time, practical applications.

## Abstract 2

Monodispersed colloidal gold nanoparticles (AuNPs) were synthesized by an easy, cost-effective, and eco-friendly method for electrochemical detection of As(iii).

## Graphical abstract

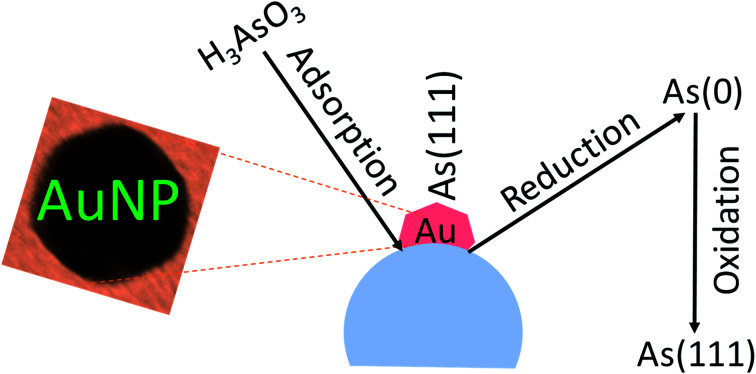

## 1 Introduction

Arsenic (As) is a toxic element, which even at trace levels can cause dangerous health effects, including death.^1–3^ Contamination of drinking water by As has been reported worldwide, threatening the health of 140 million people.^4^ Hence, the World Health Organization (WHO) and the United States Environmental Protection Agency (USEPA) have established that As concentration in drinking water must be below 10 μg L^−1^ (*i.e.*, 10 parts per billion, ppb).^5,6^ Although As exists in different forms in nature, in groundwater it exists primarily in two inorganic forms: pentavalent arsenate (As(v)), and trivalent arsenite (As(iii)). Between these two, As(iii) is more harmful with toxicity at least 60 times that of As(v) and other organic arsenic types.^7^ Depending on the pH, there are different forms of As(iii): AsO_3_^3−^ (pH 14), HAsO_3_^2−^(pH 13), H_2_AsO_3_^−^ (10 < pH < 12) and H_3_AsO_3_ (0 < pH < 9) and As(v): [AsO_4_(H_2_O)_12_]^3−^ (pH > 13), [HAsO_4_(H_2_O)_6_]^2−^ (7 < pH < 11), [H_2_AsO_4_(H_2_O)_2_]^−^ (3.5 < pH < 6) and H_3_AsO_4_ (pH < 3.5).^8^ Persistent exposure to As(iii) above the WHO and USEPA threshold value of 10 μg L^−1^ may cause a number of diseases such as skin damage, issues with circulatory systems and different cancers, including those of the skin, the lungs, the bladder and the prostate.^9^

Several analytical methods have been developed for the determination of As.^10–12^ These methods usually involve expensive and large laboratory instruments such as, surface enhanced Raman spectroscopy (SERS), atomic absorption spectroscopy (AAS), flame AAS (FAAS), graphite furnace AAS (GFAAS), and inductively coupled plasma mass spectroscopy (ICPMS).^13–15^ Consequently, simple, rapid, and onsite analytical methods for ultrasensitive determination of As are being actively developed.

Aptamers are considered as appealing tools for detecting the presence of heavy metals in the environment.^16,17^ An As-binding DNA aptamer, Ars-3, has high affinity for As(iii).^16,17^ Colorimetric and SERS methods using Ars-3 have simplified the procedure and improved the selectivity for As(iii) detection; however, exposure to As(iii) is evident at much lower concentrations than previously thought.^16,17^ A latest epidemiological study reported skin cancer cells caused by direct exposure to reduced concentrations of arsenic (10 μg L^−1^) *via* drinking water.^16,17^

Electrochemical approaches are convenient alternatives to the conventional analytical methods.^18,19^ The electrochemical methods are simple, inexpensive, user-friendly, and suitable for on-site measurements with minimal off-line preparation. Generally, cathodic and anodic stripping voltammetry (CSV/ASV) are used for the detection of As(iii) and As(v).^8,20^ These methods involve electrochemical deposition of As on an electrode for several minutes (*i.e.*, As^3+^+ 3e^−^ → As^0^) followed by their oxidation back into the solution by a reverse potential scan (*i.e.*, As^0^ → As^3+^ + 3e^−^).^21–24^ With the introduction of new pulse voltammetric techniques such as square wave voltammetry (SWV) and differential pulse anodic stripping voltammetry (DPASV), signal-to-noise enhancement through reduction in capacitance background provides considerably better detection limits than expensive spectroscopic methods.^25^

From the analytical point of view, gold electrodes are the most suitable due to their inertness and best interaction with As (*i.e.*, the formation of bimetallic compounds Au_*x*_–As_*y*_), which favor the preconcentration of trace As, thus enabling low detection limits and short analysis time.^26–28^ Plethora of various micro- or even nano-structured gold electrodes for the determination of As has been widely reviewed. Such electrodes include gold wires, gold plated microelectrodes, and conventional solid electrodes modified with gold nanoparticles (AuNPs) or AuNPs-decorated composites based on reduced graphene oxide (rGO), exfoliated graphite, magnetic nanospheres, *etc.* AuNPs provide important functions for electroanalysis: improved mass transport, sensitivity, electrocatalytic effects, and ability to adsorb metal ions. The electrochemical behavior of Au electrodes has a strong relationship with their crystallographic orientation. A single-crystal Au (111) electrode with a well-ordered surface can exhibit well-defined electrochemical behavior for As(iii) detection.^29^ Gold nanocubes (100), octahedra (111), and also rhombic dodecahedra (110) have been reported for As detection.^29^ Au (111) face was found to exhibit the highest sensitivity compared with Au (100) and (110) surfaces. The electrochemical detection of As(iii) utilizing a platform based on Au (111)-like surface by the partial reductive desorption of *n*-butanethiol (*n*-BT) was reported.^30^ A self-assembled monolayer of *n*-BT was developed, which enabled the selective blockage of Au (100) and Au (110) by *n*-BT while the Au (111) domain stayed bare. The electrode was highly sensitive and selective to As(iii) and can detect As(iii), even in the presence of high concentration of Cu(ii) with no interference. It was believed that the exposed Au (111) surface domain of the electrode played a crucial role in the detection of As(iii).^30^ Although these reported electrodes allow the detection of As at the ppb or sub-ppb levels, they have to deal with complicated fabrication methods needing instrumentation, extensive morphological control, the use of costly reagents or electroplating baths that result in high waste loadings, intensive labor and, on the whole increased cost.^26–28^ Also, interferences from various other metals such as copper (Cu), mercury (Hg), selenium (Se) that may coexist with As are considerable, which has not been appropriately addressed.^31^ For example, in the CSV evaluation, As(iii) is preconcentrated at a negative potential (−0.5 V with silver/silver chloride (Ag/AgCl)) in the presence of Cu(ii) or Se(iv) as a Cu_*x*_As_*y*_ intermetallic compound on a mercury (Hg) electrode prior to stripping.^31^ Ferreria^31^ reported interference of Cu(ii) during As(iii) measurement at Au macroelectrode and at AuNPs-modified electrodes. Copper co-deposits with As during the pre-deposition step and forms an intermetallic compound Cu_3_As_2_ and also with bulk Cu.^31^ It has been reported that making use of Au macroelectrodes, As(0) to As(iii) as well as Cu(0) to Cu(ii) stripping peaks develop within 100 mV of each other along with a third peak arising from the intermetallic compound.^31^ Deconvolution of As or even Cu peaks can be quite complicated when the metals are both present in similar amounts along with the Cu stripping peak appearing as a shoulder on the As stripping peak.^31^ If the concentration of Cu(ii) is high, then the stripping peak of Cu(ii) partially masks the As(iii) signal.

Other approaches have explored the addition of complexing agent to the electrolyte or *via* modification of Au macroelectrodes with cysteine allowing the separation of the As(111) and Cu(11) stripping peaks. On the other hand, metal ions such as Cu(ii), Hg(ii), and lead (Pb(ii)) compete for sites on the surface of Au without forming intermetallic compounds.^31^ Hence, the analytical challenge is to develop a platform for sensitive detection of trace levels of As(iii) without interference from typically co-existing heavy metals under mild condition.

Herein we report the synthesis of 15 ± 3 nm colloidal AuNPs, *via* a facile reduction method, and their use for the rapid and highly sensitive detection of As(iii). Screen-printed electrodes (SPEs) modified with the synthesized AuNPs (AuNPs/SPE) served as As(iii) sensor and was tested in both As-spiked buffer solutions and tap water. The results show that our sensor is highly sensitive, reproducible and stable. Based on this work, an electrochemical reaction mechanism for As(iii) oxidation was proposed. Interference studies, stability and repeatability measurements were conducted to assess the practicality of the sensor.

## 2 Materials and methods

### 2.1 Materials

Screen-printed electrodes were purchased from CH Instruments, Inc. (TE100, Bee Cave, TX, USA). The SPE pattern included 3 mm diameter carbon working electrode, carbon counter electrode, and Ag/AgCl reference electrode. Hydrogen tetrachloroaurate(iii) trihydrate (HAuCl_4_·3H_2_O), sodium citrate, hydrochloric acid (37%), and sodium hydroxide, were supplied by ACROS Organics. Arsenic trioxide (As_2_O_3_) was purchased from Sigma Aldrich. All other reagents were obtained either from Sigma Aldrich or Fisher Scientific with the highest grade available and were used without further purification. All solutions were prepared using deionized (DI) water with a resistivity of 18.2 MΩ cm at room temperature (Ultrapure Water System, Millipore, and Billerica, MA, USA). A 20 mM primary stock solution of As(iii) was prepared by dissolving As_2_O_3_ (solubility in water at 25 °C is 20 g L^−1^) in DI water. To produce a standard calibration curve for As(iii), different concentrations (0.001 mM, 0.01 mM, and 0.1 mM) of As(iii) solutions were prepared by diluting the primary stock solution using DI water. Tap water samples were from our laboratory at the University of Wisconsin-Madison, WI, which did not contain any visible sediments so samples were not filtered prior to use.

### 2.2 AuNPs synthesis and electrode fabrication

**Scheme 1:**
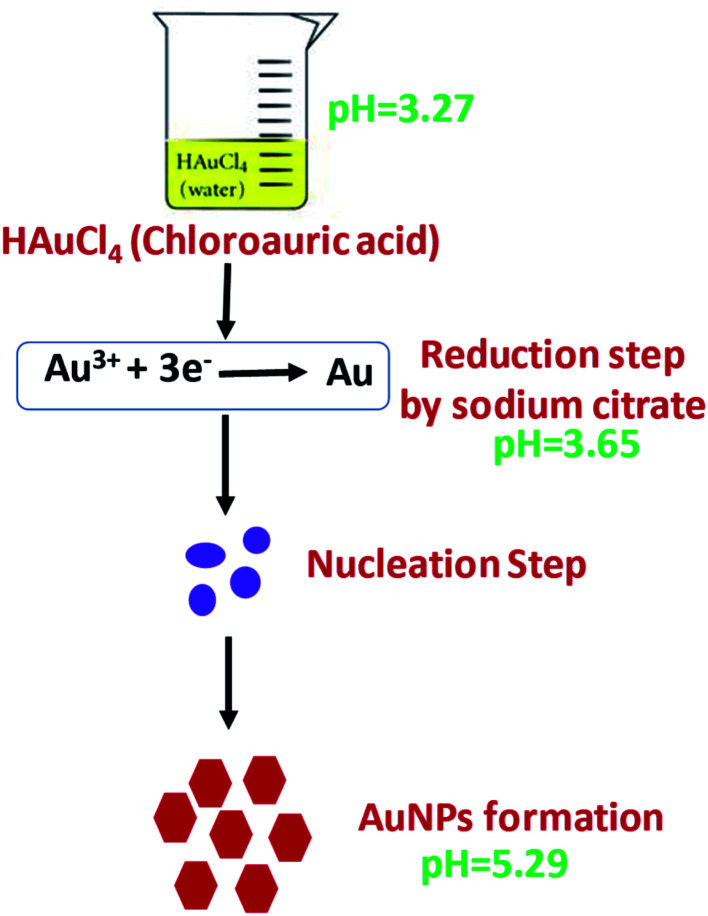
Steps in synthesis of quasi-hexagonal AuNPs.

AuNPs were synthesized according the Turkevich method,^32^ with slight modifications (Scheme 1). Two milliliters of 10 mM HAuCl_4_·3H_2_O was added to 18 mL DI water under constant stirring and the solution was brought to a boil. To this, 2 mL of 1% sodium citrate was added under stirring in an Erlenmeyer flask with stopper. The solution turned dark brown within 10 s and then to burgundy in 60 s, which signified the formation of AuNPs. The solution was cooled to room temperature and was stored in a refrigerator under dark conditions. The working electrode of SPE was modified with AuNPs by drop casting 10 μL of the colloidal AuNPs solution and allowing to air dry at room temperature.

### 2.3 Instrumentation and measurements

X-ray photoelectron spectroscopy (XPS) was performed (Thermo Scientific K Alpha instrument) to analyze the surface chemical composition and elemental distribution. High resolution transmission electron microscopy (HRTEM) images were obtained with JEOL JEM-2100F to study the morphology of the synthesized AuNPs. UV-vis absorption spectra were recorded at room temperature on a spectrophotometer (Lambda 25, PerkinElmer). Dynamic light scattering (DLS) measurements were performed to determine the average size of the AuNPs using a Nanoparticle Analyzer (NanoBrook 90Plus, Brookhaven Instruments). Electrochemical experiments were performed using CHI-660D electrochemical workstation (CHI Instruments Inc.) in the presence of 0.1 M phosphate buffered saline (PBS) solution. A micro-pipette (Eppendorf Research plus) was utilized to inject the analyte solution into PBS. To investigate the electrocatalytic behavior of the AuNPs-modified electrodes, cyclic voltammetry (CV) was performed in 1.0 M H_2_SO_4_ and also in 1.0 M NaOH at 50 mV s^−1^. The following parameters were used for performing DPASV: increment, 0.01 V; amplitude, 0.05 V; pulse width, 0.2 s; sampling width, 0.005 s; pulse period, 0.5 s; and frequency, 50 Hz. Different deposition times of 30, 60, 120, and 180 s were examined with standard additions of As(iii). With the increase of deposition time, the peak heights increased linearly. While for the detection of low concentrations of As(iii), the deposition time can be prolonged; however, for high concentrations, short deposition time should be used to avoid the saturation of the electrode surface. A deposition potential (optimized) of −0.8 V for 180 s was used for the following experiments. The assembled sensor setup is shown in Scheme S1 (ESI†).

## 3 Results and discussion

### 3.1 Role of the pH of reaction mixture

The approved mechanism of the Turkevich approach for the synthesis of AuNPs consists of the initial redox reaction (R1), where Au(iii) gets reduced to Au(i) by citrate, which in turn gets oxidized to acetone dicarboxylate (DC^2−^), the conjugated base of dicarboxyacetone (DCA). This first redox step is considered the rate-determining step. Consecutively, a disproportionation reaction (R2) occurs, where Au(0) and Au(iii) are produced (Scheme S2†).^33–36^ We have demonstrated that, the pH of the medium inevitably determines the reaction rate of the reduction, which is the rate-limiting step in the AuNPs formation and entails decarboxylation of the citrate. The mechanisms support the finding that the more acidic the medium, the faster the reduction rate. The AuNPs formation is quicker at pH = 4.7 (∼3 min to complete reaction) than at pH = 5.6 (∼5 min), and at near neutral pH = 6.5 the reaction is not complete after more than 7 min.^33–36^ This pH effect is related to the hydrolysis of the citrate and chloroauric species, since HCit^2−^ is the strongest reducing species, whose concentration is maximal at pH 5.6. Similarly, AuCl_4_^−^ is the most reactive Au precursor compared with its hydrolyzed species at high pH values (AuCl_3_(OH)^−^, AuCl_2_(OH)_2_^−^, AuCl(OH)_3_^−^ and Au(OH)_4_^−^). As a result, the reaction, especially the nucleation stage, is much faster at pH ∼3.7–6.5, compared with pH of ∼6.5–7.7.^33–36^ In our method, the R1 has a pH range of chloroauric acid solution and mixed solution to be 3.27 and 3.65, and the pH of the final solution between 5.2 and 5.4, which enabled faster reaction rate for the reduction of AuNPs compared to conventional Turkevich method.

### 3.2 Characterization of AuNPs

**Fig. 1:**
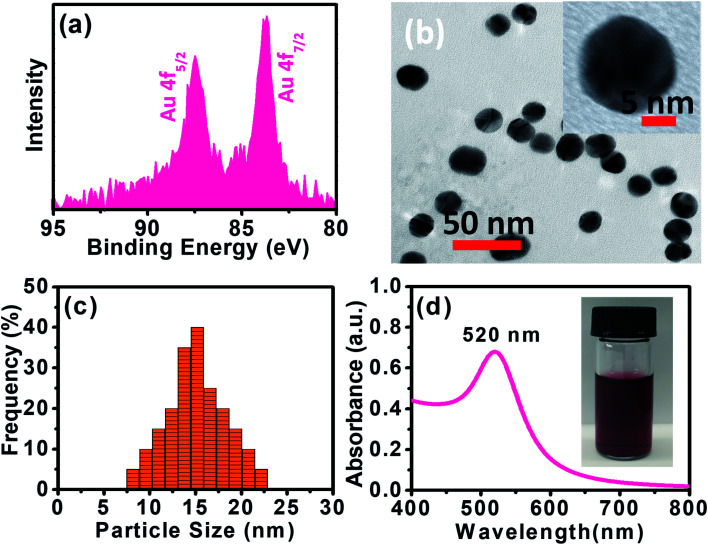
(a) XPS spectrum of the Au 4f level of AuNPs. (b) HRTEM image of Au. (c) Particle size analysis for AuNPs using DLS. (d) UV-vis spectrum of as-synthesized AuNPs. The inset is a picture of AuNPs aqueous solution measurement.

XPS data presented in Fig. 1a reveal two distinct lines due to the spin–orbit splitting of the Au 4f level.^37^ The positions of these lines, approximated after the correction due to charge accumulation, were at 87.45 eV and 83.67 eV, which correspond to Au 4f_5/2_ and Au 4f_7/2_ components, respectively.^38,39^ This clearly shows the existence of Au^0^ at 83.67 eV. The XPS scan does not display peaks corresponding to other Au valences, possibly because they are too scarce to be detected.^37^

**Fig. 2:**
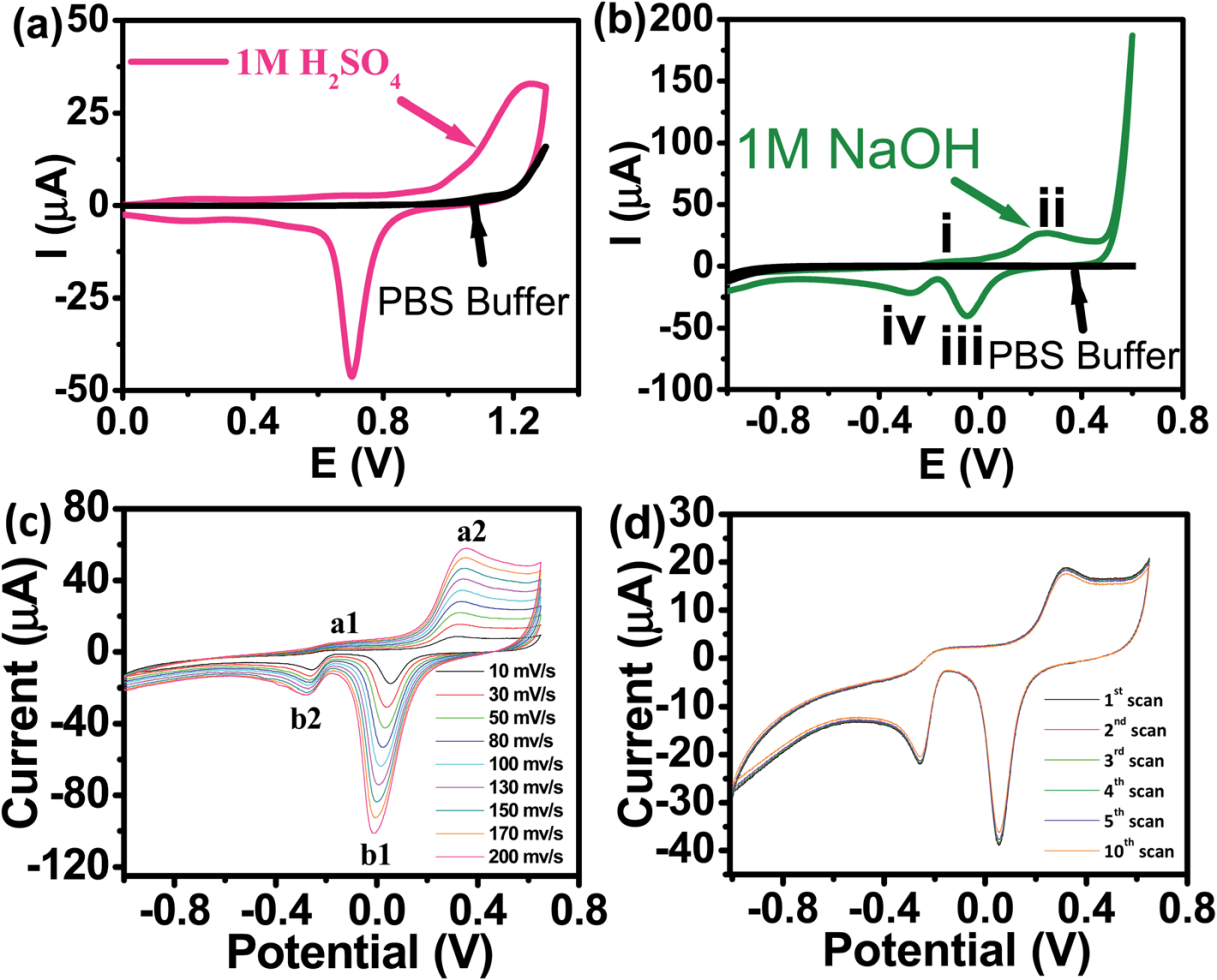
(a) CV of AuNPs/SPE in 1.0 M H_2_SO_4_ and PBS buffer (pH = 6.4) at a scan rate of 50 mV s^−1^. (b) CV of AuNPs/SPE in a 1.0 M NaOH solution and PBS buffer (pH = 6.4) at a scan rate of 50 mV s^−1^. (c) CV of AuNPs/SPE in a 1.0 M NaOH solution at scan rates from 10 to 200 mV s^−1^. (d) Cyclic stability test of AuNPs/SPE in a 1.0 M NaOH solution.

Panoramic TEM images of AuNPs samples show ∼15–18 nm crystallites of quasi-hexagonal morphology, with smaller particles exhibiting more regular shape and better dispersity (Fig. 1b). The particle size data were obtained from the TEM micrographs by measuring several particles by image processing. The TEM particle size data agreed well with the hydrodynamic diameter (∼15 nm) measured by DLS (Fig. 1c). UV-vis spectra of the AuNPs solutions showed plasmon resonance at 520 nm, which is characteristic of small (<15 nm) AuNPs (Fig. 1d).^40,41^ The CV curve of AuNPs/SPE measured in 1.0 M H_2_SO_4_ showed oxidation in the positive scan (1.2 V) with the formation of AuOH (Fig. 2a). This is followed by the formation of gold oxide monolayers, such as AuO or Au_2_O_3_. In the reverse scan, a major cathodic peak appears at 0.69 V corresponding to the reduction of gold oxide to metallic gold.^4,42^ However, in PBS electrolyte, no distinguishable current response for AuNPs/SPE was observed. The voltammogram of AuNPs/SPE in the alkaline medium (1.0 M NaOH) resembles that of a bulk gold electrode (Fig. 2b). The anodic peak (i) is because of the anodic discharge of water, with the formation of a sub monolayer of adsorbed hydroxyl radicals, while the oxidation wave (ii) is related to Au(iii) formation.^43^ The cathodic peaks (iii) and (iv) in the negative sweep are related to the reduction of Au(iii) species.^43^ However, in PBS electrolyte, no distinguishable current response for AuNPs/SPE was observed. To study the effect of bulk oxide formation and reduction on Au dissolution, CV curves at various scan rates (10–200 mV s^−1^) in 1 M NaOH at AuNPs/SPE was recorded over a wider potential region (Fig. 2c). The oxidation and reduction peaks do not shift to more positive or negative potentials for longer polarization times. Both OH adsorption/desorption, and oxide formation/reduction are reversible processes on AuNPs/SPE suggesting the stability of the electrode.^44^ The stability of the modified electrode was also tested by cycling the electrode continuously in 1 M NaOH. There was no apparent decrease in the current response for 10 consecutive cycles, whereas the peak shape unchanged, demonstrating that the surface roughness remained almost unaffected indicating that the modified electrode was relatively stable (Fig. 2d).

**Fig. 3:**
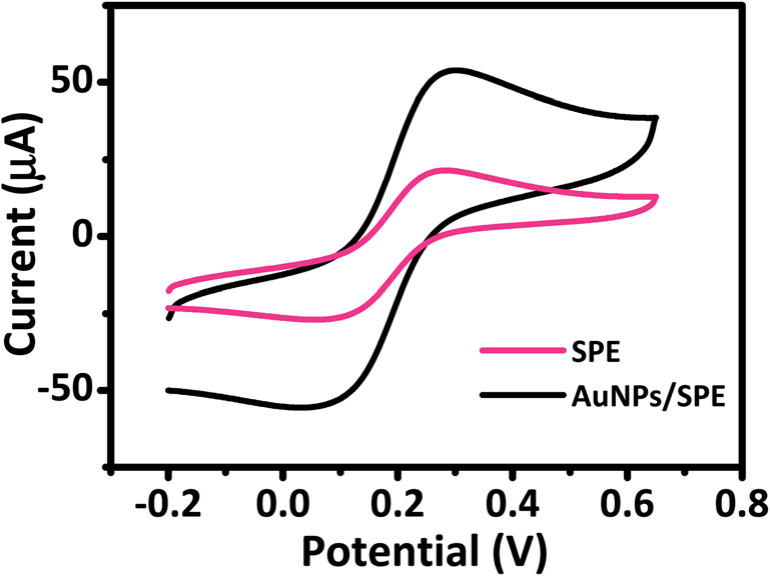
Cyclic voltammograms of prepared electrodes in 5 mM [Fe(CN)_6_]^3−/4−^ and 0.5 M KCl solution at scan rate of 50 mV s^−1^.

The electrochemical behavior of AuNPs/SPE and SPE was investigated by CV to explain their electron transfer processes in 0.5 M KCl and 5 mM [Fe(CN)_6_]^3−/4−^ solution at a scan rate of 50 mV s^−1^. CV curves in Fig. 3 show a pair of well-defined quasi-reversible anodic and cathodic peaks, where the peak current intensity (*i*_p_) increases remarkably for AuNPs/SPE compared with SPE. These changes are due to the higher active surface area of AuNPs/SPE than that of bare SPE. We used the Randles–Sevcik equation to estimate the active surface area of the electrodes.^45,46^*i*_p_ = 2.69 × 10^5^*AD*^1/2^*n*^3/2^*v*^1/2^*C*where, *n* = number of electrons participating in the redox reaction, *A* = electroactive area (cm^2^), *D* = diffusion coefficient of the bulk concentration of the redox probe (cm^2^ s^−1^), *C* = concentration of the probe molecule in the bulk solution (mol cm^−3^), *v* = scan rate (V s^−1^). The [Fe(CN)_6_]^3−/4−^ is one of the most extensively studied redox couples in electrochemistry and exhibits a heterogeneous one-electron transfer (*n* = 1). For this study, reported value for *C* = 5 mM, *D* = 6.7 × 10^−6^ cm^2^ s^−1^.^45,46^ The calculated electroactive surface area for AuNPs/SPE (0.096 cm^2^) is almost three times that of the bare SPE (0.033 cm^2^) due to the incorporation of AuNPs.

### 3.3 Effect of pH

**Fig. 4:**
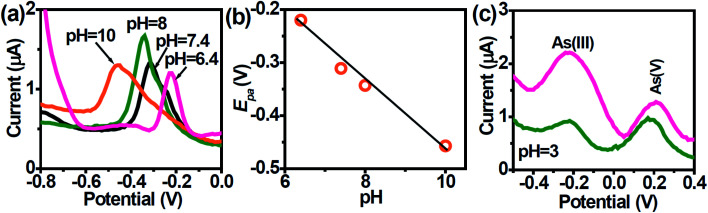
(a) DPASV at AuNPs/SPE in PBS buffer solution at different pHs in the range of 6.4 to 10. (b) Dependence of the peak potentials with pH. (c) DPASV with varying concentrations of AS(iii) and As(v) ions obtained with the AuNPs/SPE (1 and 50 nM).

The voltammetric behavior of heavy metal ions is strongly influenced by the pH of the supporting electrolyte and thus it was essential to select a suitable pH value. The speciation of As is pH-dependent, which will impact its quantification. As(iii) exists predominantly within the pH region 2–8 to its non-ionic form *i.e.*, H_3_AsO_3_ which gradually turns to anionic species of H_2_AsO_3_^−^ beyond pH 8.^47^ At pH above 7, ionization occurs leading to the formation of anionic H_2_AsO_3_^−^ (pH 7.0–8.0),^47^ and HAsO_3_^2−^ at pH > 10.^12,48^ The peak potentials shift negatively with increasing pH between 6.4–10 (Fig. 4a) due to changes in As speciation.^47^ A plot of peak potential *vs.* pH show a linear relationship with a slope value of ∼60 mV pH^−1^ which is close to the Nernstian value indicating the number of electrons and protons taking part in the electrochemical reaction was the same (Fig. 4b).^49^

1 H_3_AsO_3_ + 3H^+^ + 3e^−^ → As + 3H_2_O

2 *E* = *E*^0^ + (0.0591/*n*) log([H_3_AsO_3_] [H^+^]^3^)

When pH increases, the stripping peak potential decreases. The large overpotential of the stripping peak in alkaline condition (pH 10.0) is due to the formation of hydrolyzed species during stripping step.

As(v) is generally considered electrochemically inert under normal conditions, but can be directly electro-reduced to As(0) at pH below 3.5. The electrochemistry of As(iii) and As(v) at the AuNPs/SPE was examined at pH 3.0 (Fig. 4c). Given that the As electrochemistry is pH-dependent, two well-resolved anodic peaks were obtained. According to Smedley and Kinniburgh,^50^ it is possible to have both As(iii) and As(v) species present at harsh acidic condition. Since the larger peak at ∼ −0.2 V is attributed to As(iii), the second peak at ∼ +0.2 V can be attributed to As(v). Though for results in Fig. 4c, As(v) was not spiked into the test sample, we suggest that in the DPASV mode, it is possible to selectively determine trace amounts of As(v), though at extremely harsh acidic pH.

### 3.4 Electrochemical detection of As(iii)

**Fig. 5:**
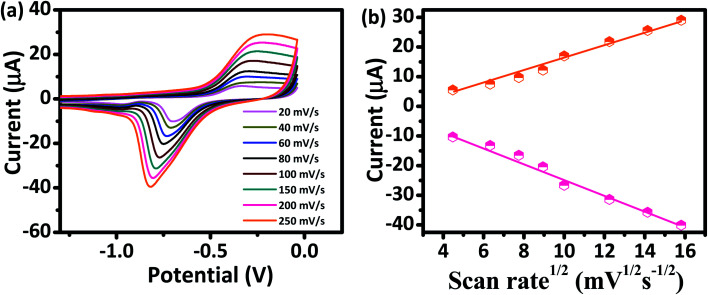
(a) CV responses of AuNPs/SPE at scan rates from 20 to 250 mV s^−1^ in PBS buffer (pH = 6.4) for As(iii) (200 nM). (b) Oxidation and reduction peak currents against the square root of scan rate.

**Fig. 6:**
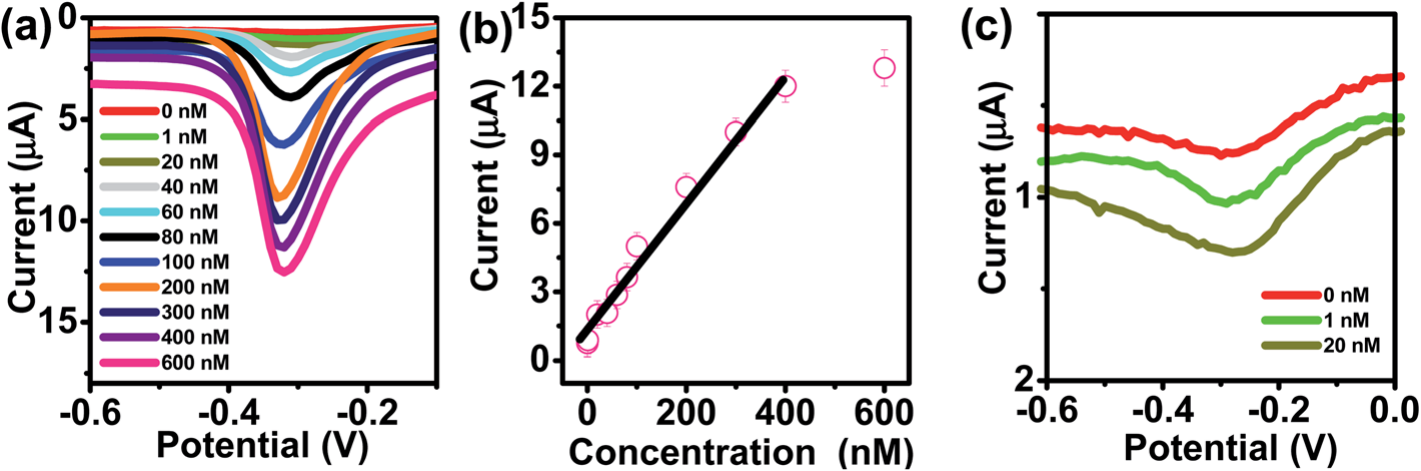
(a) DPASV with varying concentrations of As(iii) at AuNPs/SPE (pH = 6.4). (b) The calibration curve. (c) Enlarged view of low concentration (0, 1, and 20 nM).

**Scheme 2:**
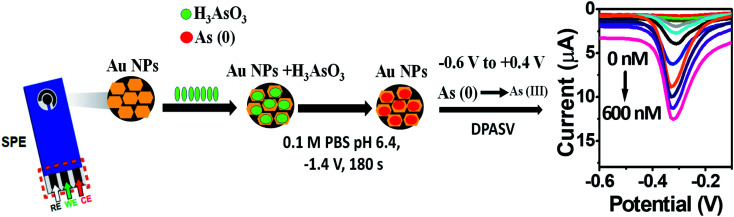
Electrochemical sensing of As(iii) on AuNPs/SPE.

The kinetics of electrode reaction was investigated *via* CV by evaluating the effect of scan rate on the oxidation and reduction peak currents of As. A pair of well-defined redox peaks corresponding to the reduction of As^3+^ to As^0^ and an oxidation peak corresponding to the oxidation of As^0^ to As^3+^ are obtained with AuNPs/SPE (Fig. 5a). The anodic and cathodic peak currents increased with scan rates and had a highly linear relationship with the square root of scan rates (20 to 250 mV s^−1^) (Fig. 5b), which indicates that the electrode reaction is diffusion controlled and hence the electrode is well-suited for quantitative measurements.^51,52^ The DPASV data at various As(iii) concentrations obtained with the AuNPs/SPE sensor are shown in Fig. 6a. The first step in electrochemical detection of As is pre-concentration of H_3_AsO_3_ at the AuNPs/SPE surface from the bulk solution (Scheme 2). The second step involves the reduction of As species to As(0) at −0.8 V followed by its stripping (reoxidation of As(0) to As(iii)).^53–57^ The characteristic peak for As(iii) was observed at ∼ −0.31 V. It should be noted that the predominant As species in water at pH 6.4 is H_3_AsO_3_. To maximize the detection, various experimental parameters (type and pH of supporting electrolyte, deposition potential, and deposition time) were optimized. As can be observed in Fig. 6a and b, the current peak at −0.31 V increases with increasing As(iii) concentration. The calibration plot of As(iii) concentration (*C*) *vs.* peak current (*I*) (Fig. 6b) was linear over the concentration range of 0.075–30 μg L^−1^ (1–400 nM) and can be described by the following regression equation:*I* (μA) = 1.2534 + 0.0285*C* (nmol L^−1^) (*R*^2^ = 0.9838)The slope of this equation represents detection sensitivity of 28.5 nA nM^−1^.

The limit of detection (LOD) and limit of quantification (LOQ) are defined as “the lowest amount of analyte in a sample which can be detected but not necessarily quantitated as an exact value” and “the lowest amount of analyte in a sample which can be quantitatively determined with suitable precision and accuracy”.^58^ They were calculated as follows (see Fig. S1, ESI†):
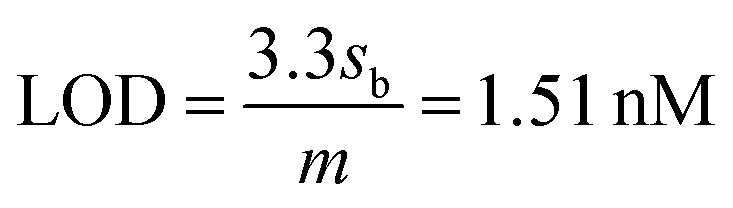

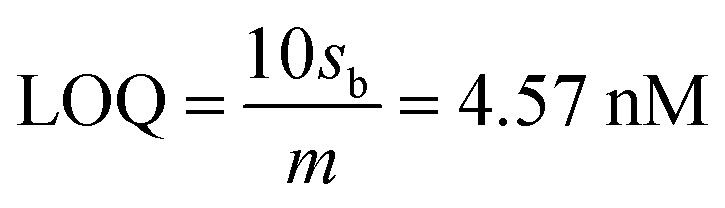
where, *s*_b_ is the standard deviation of the blank measurements and *m* is the slope of the linear calibration plot of analyte measurements. The LOQ and LOD obtained using the AuNPs/SPE system are well below the regulatory As limit of 10 μg L^−1^ established by USEPA. The very low LOQ of the proposed sensing system presents possibilities for onsite monitoring of trace quantities of As(iii). These LOD and LOQ values compare vary favorably with many other reported values for As(iii) measurement (Table S1, ESI†).^67–76^

### 3.5 Interference study

**Fig. 7:**
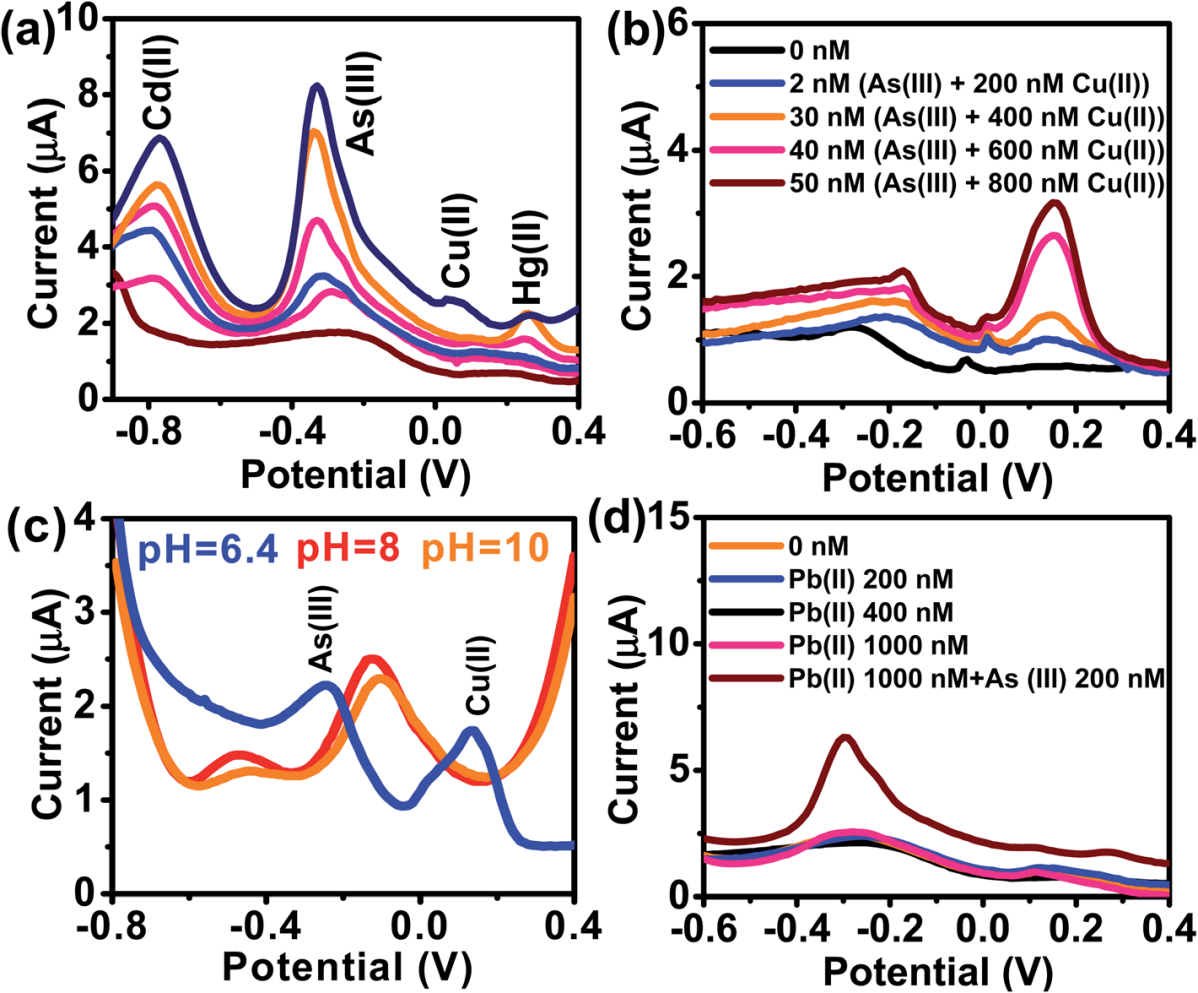
(a) DPASV responses of AuNPs/SPE in PBS buffer (pH = 6.4) shows the simultaneous detection of Cd(ii), Cu(ii), and Hg(ii) all at 0, 100, 200, 400, 600, and 1000 nM and As(iii) (0, 10, 50, 100, 150, and 200 nM). (b) DPASV responses of AuNPs/SPE in PBS buffer (pH = 6.4) shows the simultaneous detection of binary mixture of Cu(ii) and As(iii). (c) DPASV responses showing simultaneous detection of binary mixture of Cu(ii) and As(iii) at different pHs in the range of 6.4 to 10. (d) DPASV responses of AuNPs/SPE in PBS buffer (pH = 6.4) shows the simultaneous detection of Pb(ii) at 200, 400, and 1000 nM and As(iii) (200 nM).

Reliable detection of ultratrace As(iii) levels in the real-world samples (*e.g.*, tap water) without interference is a challenging task, as the other (interfering) metal ions present in the real samples can co-precipitate and strip off during As(iii) detection. Therefore, we tested our electrode to detect As(iii) ranging from 0–15 μg L^−1^ (0 to 200 nM) in the presence of Cu(ii), Hg(ii), and Cd(ii) at concentrations ranging from 0–75 μg L^−1^ (0–1000 nM) (Fig. 7a). DPASV data show that the anodic peak responses for the quaternary mixture containing Cd(ii), As(iii), Cu(ii), and Hg(ii), are well separated from each other with a potential difference of 442 mV, 380.5 mV, and 212.5 mV for Cd(ii)–As(iii), As(iii)–Cu(ii) and Cu(ii)–Hg(ii), respectively, which is large enough to simultaneously determine the individual elements in their mixture solution. It is well-known that Cu(ii) is a major interferent of As(iii)^59–61^ due to the formation of intermetallic compounds such as Cu_3_As_2_.^59,62^ However, the stripping peak potential of As(iii) (−0.31 V) is well separated from that of Cu(ii). The voltammograms for the binary mixture of As(iii) and Cu(ii) were also well separated from each other with a potential difference of Δ*E*_As(iii)–Cu(ii)_ = 305 mV. Similarly, well separated potentials were determined for Cd(ii) and Hg(ii) (Fig. 7b) and, hence, minimal interference issues due to the co-occurrence of these ions can be expected during As(iii) measurement.

The effect of pH of the supporting electrolyte on the simultaneous detection of As(iii) and Cu(ii) was also investigated over a wide pH range (6.8–10) *via* DPV with solutions containing 50 nM of As(iii) and 500 nM of Cu(ii) (Fig. 7c). At all tested pH values, the peak potential of both As(iii) and Cu(ii) were well separated, with potential shifting toward right at acidic pHs and toward left at basic pHs. Likewise, there was no interference from Pb(ii) (Fig. 7d) or several anions (1 mM each of Cl^−^, SO_4_^2−^, and NO_3_^−^).

### 3.6 Analytical application

**Fig. 8:**
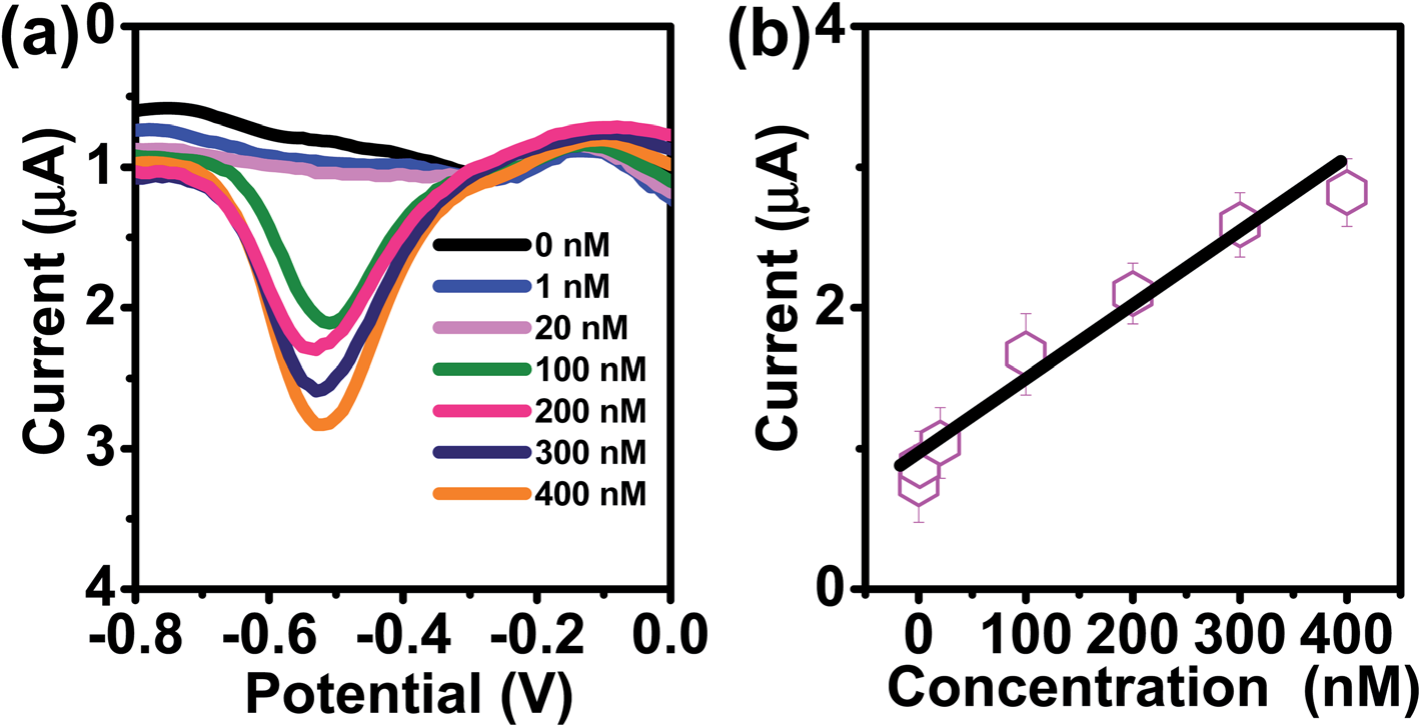
(a) DPASV with varying concentrations of As(iii) at AuNPs/SPE in tap water (pH = 7.8). (b) The calibration curve.

To evaluate the feasibility of practical applications of the AuNPs/SPE system, we performed tests for As(iii) in tap water (pH 7.4), as tap water presents a realistically complex matrix containing various metals (Al, Fe, and Mn), organic matter, and other contaminants. However, it was seen that there was no Cu, As, Zn, Cr, or Pt (Fig. 8). To determine the sensitivity and linear range of the sensor, DPASV responses are displayed in Fig. 8. As shown in (Fig. 8a), the current peak at −0.51 V increases with increasing As(iii) concentrations. The calibration plot for As(iii) was linear over the range of 0.075 μg L^−1^ −30 μg L^−1^ (1–400 nM) according to: *I* (μA) = 0.93 + 0.052*C* (nmol L^−1^)** **(*R*^2^ = 0.9652), with sensitivity (0.052 μA μM^−1^) as shown in Fig. 8b. The LOQ was 0.075 μg L^−1^ (1 nM) at 180 s deposition time and 60 s stripping time with the theoretical LOD of 0.038 μg L^−1^ (0.51 nM). This LOQ and LOD obtained using the AuNPs/SPE is well below the 10 μg L^−1^ USEPA regulatory limit.

### 3.7 Mechanistic study of As(iii) detection at the AuNPs/SPE

**Fig. 9:**
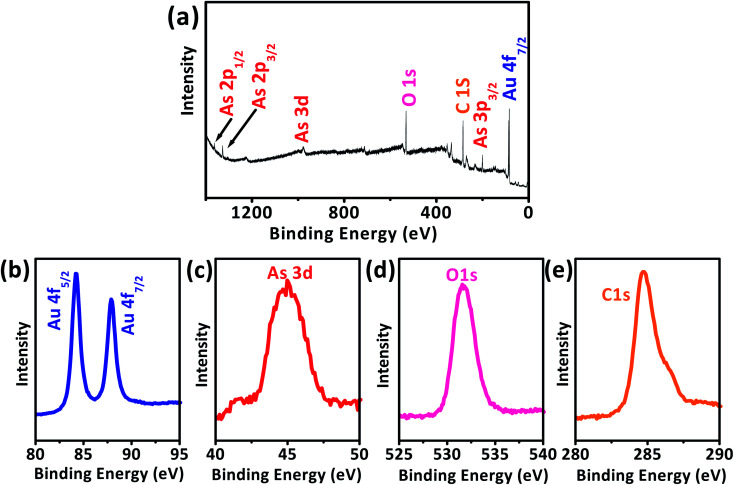
XPS spectra for the As(iii) detection at AuNPs/SPE (a), high-resolution spectra for all elements (b) Au 4f, (c) As 3d, (d) O 1s, and (e) C 1s.

The AuNPs/SPE that was used for As(iii) detection was characterized to further confirm the deposition of As(iii) and AuNPs on the SPE working surface. While applying more negative potential can induce reduction of As(iii) of the non-ionic arsenic (H_3_AsO_3_), there is a strong relation between electrical conductivity and adsorption of As(iii), *i.e.*, As(iii) adsorption ability increases with increase in conductivity of the nanomaterial.^59–61^ The XPS survey spectrum of As(iii) detection at AuNPs/SPE electrode reveals multiple regions of Au 4f, As 3d, O 1s, and C 1s (Fig. 9a),^63–66^ for which their accumulated amount *versus* binding energy spectra are shown in Fig. 9b–e. Two unique peaks separated by 3.78 eV were observed in Fig. 9b for Au 4f_5/2_ and Au 4f_7/2_ are attributable to the spin–orbit splitting of the Au 4f level.^63,64^ A clear As 3d peak at the binding energy of 44.9 eV is due to the presence of arsenic on the electrode (Fig. 9c). The O 1s peak Fig. 9d at binding energy of 531.4 eV are designated to oxygen in As_2_O_3_ and the C 1s peak in Fig. 9e is from carbon in the SPE. On the whole, XPS evaluation validates the presence of As_2_O_3_ at the AuNPs/SPE surface, because the average binding energy values of As_2_O_3_ (3d_5/2_) is 45 eV and that of As_2_O_5_ (3d_5/2_) is above 46 eV, which clearly indicates that As(iii) was detected at the electrode surface and not As(v).^63,64^

### 3.8 Stability and reproducibility

The reproducibility and stability of the sensor were evaluated. Three AuNPs/SPE modified electrodes were made and their current responses to 200 nM As(iii) was investigated. The relative standard deviation (RSD) was found to be 1.1%, confirming that our electrode fabrication method was highly reproducible. The long-term stability of the sensor was evaluated by measuring its sensitivity to 200 nM As(iii) solution for seven days. The sensor was stored at room temperature. The DPV response of the electrode to the same concentration of As(iii) decreased less than 4.0% indicating that the electrode has good reproducibility and excellent long-term stability.

## 4 Conclusions

A facile and inexpensive approach for scalable synthesis of colloidal AuNPs is described. Further, we demonstrated the usability of a AuNPs as a catalyst for electrochemical detection of As(iii). The LOQ and LOD obtained using AuNPs/SPE were 0.34 μg L^−1^ (4.57 nM) and 0.11 μg L^−1^ (1.51 nM), respectively. AuNPs/SPE sensing system facilitates As(iii) detection at concentrations around 1 nM. The sensor response is free of interference from common co-existing heavy metals such as Cd(ii), Cu(ii), and Hg(ii), which can also be simultaneously detected in a mixed solution without medium exchange or activation. AuNPs/SPE shows individual well-defined voltammetric peaks for As(iii), Cu(ii), Cd(ii), and Hg(ii). In addition, we successfully tested the sensor for detecting As(iii) in tap water, in which the linear range of detection was from 0.075–30 μg L^−1^.

## Abbreviation

HMDE: Hanging mercury drop electrode
NP: Nanoparticle
GCE: Glassy carbon electrode
BDD: Boron-doped diamond
PANI: Polyaniline
ASV: Anodic stripping voltammetry
SWASV: Square wave anodic stripping voltammetry
DPASV: Differential pulse anodic stripping voltammetry
LSV: Linear sweep voltammetry

## Conflicts of interest

There are no conflicts to declare.

## Supplementary Material

RA-010-D0RA02750B-s001
