# Supplementary material for: Ultrathin quasi-hexagonal gold nanostructures for sensing arsenic in tap water
Source: RSC Adv. 2020 May 27;10(34):20211–21. doi: 10.1039/d0ra02750b (PMC9059146; doi:10.1039/d0ra02750b)
Supplement: RA-010-D0RA02750B-s001 [file RA-010-D0RA02750B-s001.pdf]

Electronic Supplementary Material (ESI) for RSC Advances.
This journal is © The Royal Society of Chemistry 2020

Supporting Information

Ultrathin Quasi-hexagonal Gold Nanostructures for Sensing Arsenic in Tap Water

Anu Prathap M. Udayan‡

Batul Kachwala‡

K. G. Karthikeyan

Sundaram Gunasekaran*

Department of Biological Systems Engineering

University of Wisconsin–Madison, WI 53706, USA

* Corresponding author (Email address: guna@wisc.edu)

‡These authors contributed equally

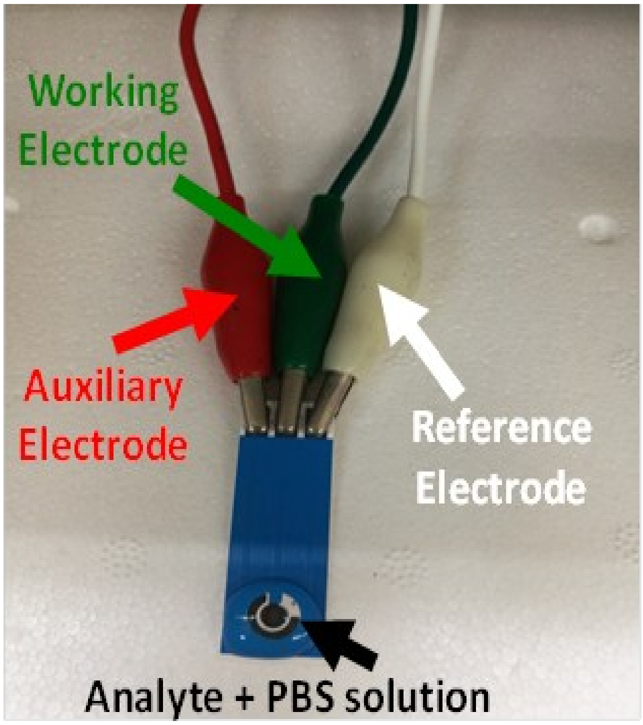

Scheme S1. Photograph of the entire experimental set up.

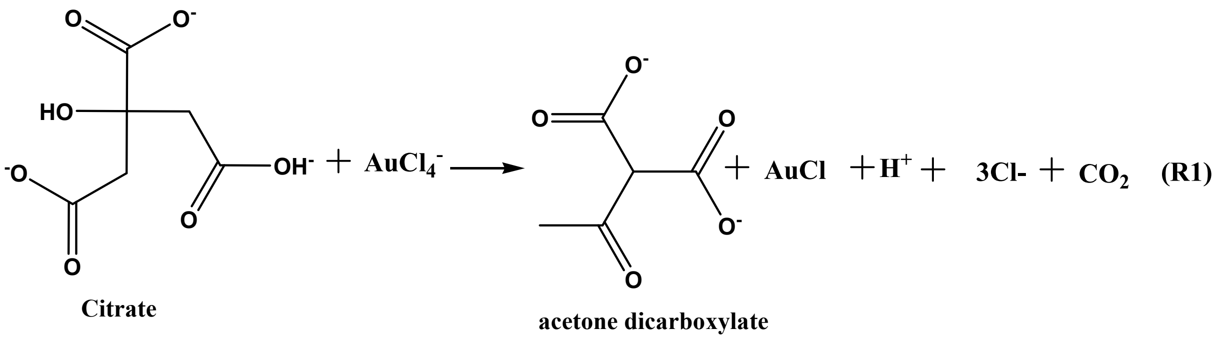

$$3AuCl + Cl^- \longrightarrow 2Au^0 + AuCl_4^- \quad (R2)$$

Scheme S2. The accepted mechanism for Turkevich method.

Calculation of limit of detection (LOD) and limit of quantification (LOQ):

$$\mathbf{LOD = 3.3*s_b/m} \quad \text{and} \quad \mathbf{LOQ = 10*s_b/m}$$

Where, s_b is the standard deviation of the blank sample measurement and m is the slope of the linear calibration plot of analyte measurement[1].

Calculation of slope m:

As (III) concentration (nM)	Current (μA)
0	0.75
1	0.88
20	2.0
40	2.07
60	2.86
80	3.64
100	5.0
200	7.59
300	10.0
400	12.0

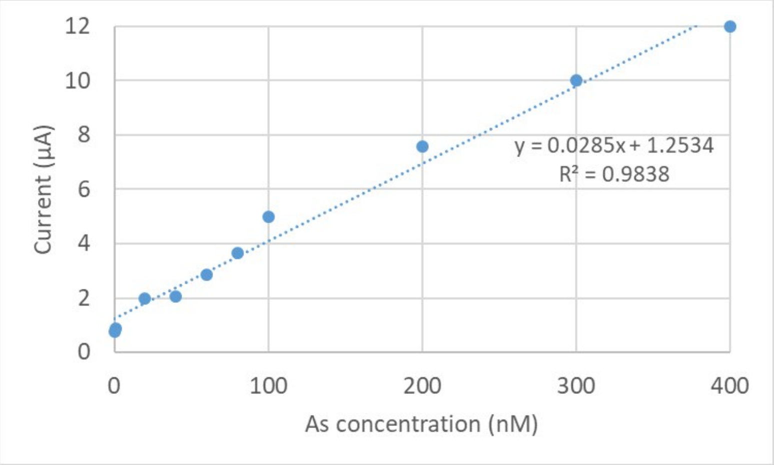

Slope, m = 0.0285

Calculation of standard deviation of blank S_b:

Standard deviation (SD) formula:

$$s = \sqrt{\frac{\sum_{i=1}^{n}\left(x_i - \bar{x}\right)^2}{n-1}}$$

Blank sample	Current value of blank solution (μA)
x_1	0.75
x_2	0.78
x_3	0.76
x_4	0.77
x_5	0.75
Statistics	Mean, $\bar{x}$=0.76
	s_b=0.013038

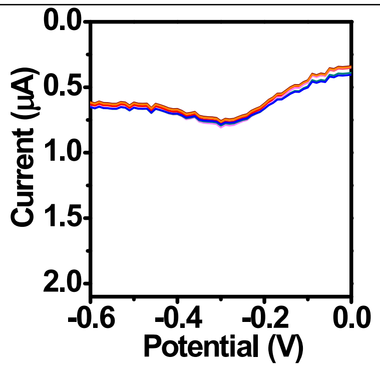

LOD = $3.3*s_b/m$**=** (3.3*0.013038)/0.0285 = 0.0391/0.0285 = **1.51 nM**

LOQ = (10*0.013038)/0.0285 = 0.13038/0.0285 = **4.57 nM**

Table. S1. Comparison of published electrochemical As(III) sensors with that in the present work

Electrode material	Voltammetry	Tested sample	Electrolyte used	LOD (nM)	LOQ (nM)	Interferents	Reference
Au plated carbon paste	Constant current Stripping Analysis	Contaminated freshwater	0.25 M HCl + 1.25 M $HClO_4$	40	-	Cu	[2]
HMDE	SWCSV	Spiked freshwaters	1 M HCl + 45 ppm Cu(II)	0.8	2.7	Cu, Sb	[3]
Au graphite	ASV	-	EDTA-H_3PO_4	-	174	30XCu	[4]
Au coated BDD thin-film	DPASV	Contaminated freshwater	Cation exchange resin/ 1 M HCl	0.067	0.13	Cu	[5]
Au film	Derivative anodic stripping chronopotentiometry	Wine sample	3 M HCl	1	3.4	10X Cu	[6]
AuNP on GCE	LSV	-	1 M HCl + 2 µM Cu(II)	0.08	-	Cu	[7]
Silver	SWASV	-	0.1 M HNO_3	14	-	-	[8]
Au coated BDD thin-film	DPASV	Contaminated freshwater	Cationexchange resin / 1 M HCl	0.067	0.13	Cu	[9]
AuNP/PANI-modified GCE	SWASV	-	1 M HCl	5.4	-	-	[10]
Pt nanotubes arrays on GCE	LSV	Contaminated freshwater	0.1 M H_2SO_4	1.3	13.4	Cl	[11]
AuPd NP on GCE	SWASV	-	0.1 M acetate buffer	0.33	13.4	Cu	[12]
AuNP on carbon polyethylene composite	LSV	Spiked drinking water	0.1 M Na_2SO_3	1.2	-	Cu	[13]
AuNPs/SPE	DPASV	Spiked drinking water	PBS	1.51 nM	4.57	None	**This study**

References

1 ICH, *ICH Topic Q 2 (R1) Validation of Analytical Procedures: Text and Methodology*, European Medicines Agency, 1995.

2 I. Svancara, K. Vytras, A. Bobrowski and K. Kalcher, *Talanta*, 2002, **58**, 45–55.

3 M. A. Ferreira and A. A. Barros, *Analytica Chimica Acta*, 2002, **459**, 151–159.

4 A. I. Kamenev, A. B. Lyakhov and S. E. Orlov, *J Anal Chem*, 2005, **60**, 156–162.

5 Y. Song and G. M. Swain, *Anal. Chim. Acta*, 2007, **593**, 7–12.

6 G. Dugo, L. La Pera, V. Lo Turco and G. Di Bella, *Chemosphere*, 2005, **61**, 1093–1101.

7 X. Dai and R. G. Compton, *Electroanalysis*, 2005, **17**, 1325–1330.

8 A. O. Simm, C. E. Banks and R. G. Compton, *Electroanalysis*, 2005, **17**, 1727–1733.

9 Y. Song and G. M. Swain, *Anal. Chim. Acta*, 2007, **593**, 7–12.

10 X. Dai, G. G. Wildgoose, C. Salter, A. Crossley and R. G. Compton, *Anal. Chem.*, 2006, **78**, 6102–6108.

11 H. Xu, L. Zeng, S. Xing, G. Shi, J. Chen, Y. Xian and L. Jin, *Electrochemistry Communications*, 2008, **10**, 1893–1896.

12 Y. Lan, H. Luo, X. Ren, Y. Wang and Y. Liu, *Microchim Acta*, 2012, **178**, 153–161.

13 E. A. Zakharova, G. N. Noskova, S. G. Antonova and A. S. Kabakaev, *International Journal of Environmental Analytical Chemistry*, 2014, **94**, 1478–1498.
